# Tissue Destruction Caused by *Entamoeba histolytica* Parasite*:* Cell Death, Inflammation, Invasion, and the Gut Microbiome

**DOI:** 10.1007/s40588-019-0113-6

**Published:** 2019-01-21

**Authors:** Swagata Ghosh, Jay Padalia, Shannon Moonah

**Affiliations:** 1 Department of Medicine, University of Virginia School of Medicine, Charlottesville, VA USA; 2 Division of Infectious Diseases, Department of Medicine, University of Virginia Health System, 345 Crispell Drive, MR-6 1st floor, Room 1709, Charlottesville, VA 22908 USA

**Keywords:** Amebiasis, Trogocytosis, Microbiome, Invasion, *E. histolytica* macrophage migration inhibitory factor

## Abstract

**Purpose of Review:**

*Entamoeba histolytica* is a protozoan parasite that causes amebiasis, which remains a significant cause of morbidity and mortality worldwide. *E. histolytica* causes tissue destruction which leads to clinical disease. This review outlines some of the recent advances that have furthered our understanding of the processes that lead to the tissue damage caused by *E. histolytica.*

**Recent Findings:**

Recent studies have identified new mechanisms involved in *E. histolytica*–induced tissue damage. These include (i) new form of contact-dependent killing called trogocytosis; (ii) parasite-produced cytokine, macrophage migration inhibitory factor, that contributes to inflammation; (iii) exploitation of host immune response to promote invasion; and (iv) the contribution of the gut microbiome to clinical disease.

**Summary:**

Targeting these mechanisms that result in tissue injury should be a focus of future research for the development of improved preventive and therapeutic strategies for amebiasis.

## Introduction

*Entamoeba histolytica* is a pathogenic protozoan parasite that causes amebiasis in humans. *E*. *histolytica* infection can be asymptomatic or lead to severe disease with amebic colitis and amebic liver abscess. Amebiasis remains a significant cause of morbidity and mortality worldwide. *E. histolytica* infection is estimated to kill more than 55,000 people each year [1••]. Globally, diarrheal disease is the third leading cause of death in children under 5 years of age with amebic colitis being a leading cause of severe diarrhea in low-income countries [1••]. Fulminant amebic colitis is an uncommon but life-threatening complication, and on average, more than 50% with severe colitis die [2•]. *E*. *histolytica* infection is also a concern among returning travelers with infectious gastrointestinal disease, with an incidence of 14/1000 unwell travelers [3]. Nitroimidazoles are the only treatment for invasive amebiasis. Nitroimidazoles are toxic drugs, and resistance to them has developed in other anaerobic protists [1••]. Also, efforts to prevent disease are complicated by the lack of a vaccine. Therefore, it is crucial to understand amebiasis pathogenesis as a way forward to better approaches for treatment and prevention (Table 1).

**Table 1:** Key points

*E. histolytica*–induced tissue damage is a result of 3 main events: host cell death, destructive inflammatory response, and parasite invasion.	
Amebic trogocytosis is a recently discovered mechanism for host cell killing.	
A specific parasite-encoded cytokine, *E. histolytica* macrophage migration inhibitory factor (*Eh*MIF), mediates inflammation during amebiasis.	
*E. histolytica* exploits the host inflammatory response to promote tissue invasion.	
Gut microbiome influences the clinical outcomes of *E.histolytica* infection.	

**Fig. 1:**
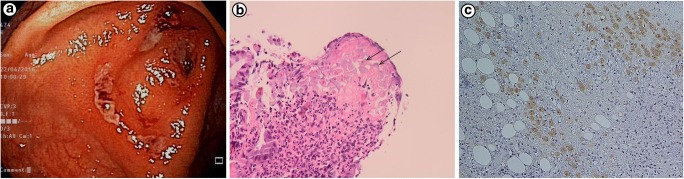
Patient with amebic colitis. **a** Intestinal ulcers due to *E. histolyica.***b***E. histolyica* within the lamina propria (arrows) surrounded by inflammatory infiltrate of neutrophils. **c** Invasion of intestinal mucosa by amebic trophozoites. Immunohistochemical staining of trophozoites (brown) using specific anti–*Entamoeba histolytica* macrophage migration inhibitory factor antibodies. Panels (a, b) are reproduced from [4] and (C) from [1••] with permission

*E. histolytica* parasite exists in two forms, a cyst stage and a trophozoite stage. The life cycle of *E. histolytica* begins with the ingestion of the infectious cyst from fecally contaminated food or water or through oral-anal sexual practices. The cysts pass through the stomach and small intestine where they excyst and form invasive trophozoites in the lumen of the intestine. Trophozoites can penetrate the mucus layer of the large intestine. Disease outcomes, namely colitis and liver abscess, are associated with vast tissue damage. For example, amebic colitis is characterized by colonic ulcers with invasion of parasites into the lamina propria and an infiltration of inflammatory cells such as neutrophils [4] (Fig. 1).

**Fig. 2:**
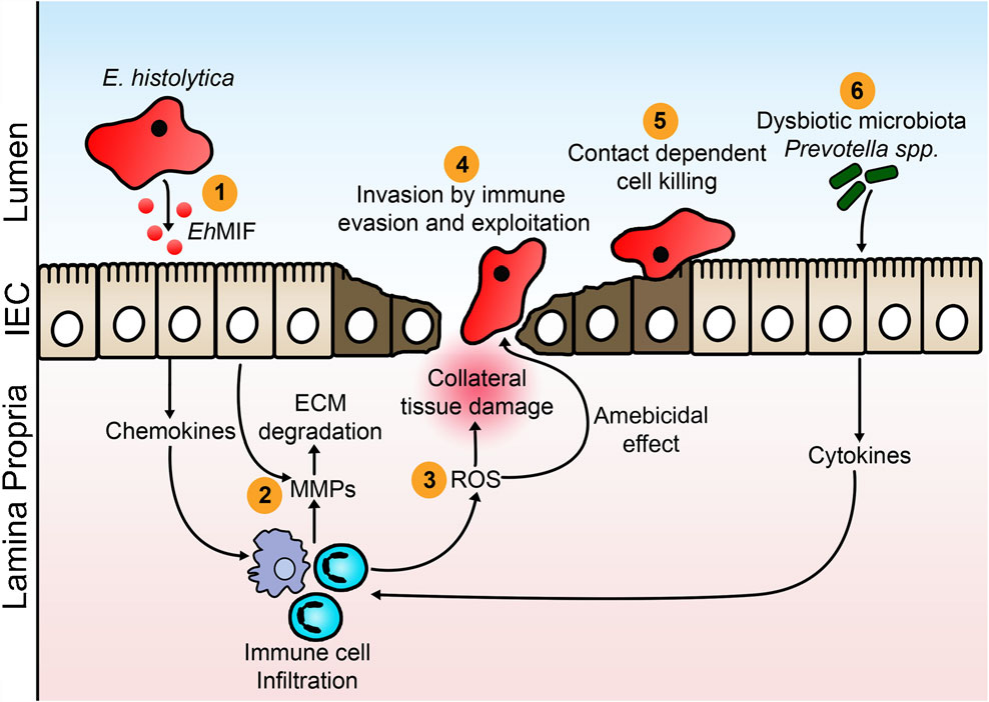
Pathogenesis of intestinal amebiasis. 1. Secreted *E. histolytica* macrophage migration inhibitory factor *(Eh*MIF) promotes mucosal inflammation. 2. *E. histolytica*–induced inflammation results in increased production in matrix metalloproteinases (MMPs) which break down extracellular matrix (ECM) in the gut to promote cell migration. 3. Infiltrating inflammatory cells generate oxygen free radicals (ROS) which are capable of killing parasites. Oxygen free radicals are also responsible for collateral tissue damage during the inflammatory period. 4. *E. histolytica* invades the intestinal mucosa by evading and exploiting the host immune system. 5. Contact-dependent cell killing by *E. histolytica*. 6. Elevated levels of *P. copri* increases the risk of colitis

Tissue damage caused by *E. histolytica* is a result of three main events: host cell death, inflammation, and parasite invasion. This report reviews recent literature expanding our knowledge of the pathogenesis of amebiasis (Fig. 2).

## Cell Death

*E. histolytica* has the remarkable ability of direct killing of host cells. The mechanism of cell killing by *E. histolytica* has been the subject of intensive investigation for several decades. Adhesion to host cells is a critical factor for the killing activity of *E. histolytica*. Gal/GalNAC lectin is the major parasite adherence molecule that is required for the contact-dependent cell killing by *E. histolytica*. The process of cytotoxicity primarily involves programmed cell death (apoptosis) as demonstrated by TUNEL and annexin V staining showing DNA fragmentation and membrane blebbing. Several independent studies have shown that caspase-3 activation leads to the induction of programmed cell death [5], though the pathway that mediates this response is not fully understood. One theory is that parasite contact causes elevation of intracellular calcium in host cells, which ultimately leads to global protein tyrosine dephosphorylation and activation of caspase-3 [6].

What are the parasite cytotoxic effector molecules that trigger host cell death? It has been proposed that *E. histolytica* secretes proteins, such as amoebapores, that have cytotoxic effects on human cells. However, this theory is challenged by the failure of *E. histolytica*–secreted fractions to kill host cells.

*E. histolytica* is also capable of ingesting host cells by a process of phagocytosis. Phagocytosis is believed to play a crucial role in amebic pathogenesis as well as nutrient uptake [7]. In vitro studies have shown that *E. histolytica* is capable of ingesting a variety of host cells. However, *E. histolytica* trophozoites obtained from clinical samples, including colonic ulcer biopsy, mainly consist of one cell type which is ingested red blood cells [1••].

More recently, real-time observation of cell killing by *E. histolytica* revealed that the parasite, prior to cell death, ingests distinct “bites” of live host cells by a process called “trogocytosis” [8••]. This leads to intracellular Ca^2+^ elevation and loss of membrane integrity in the host cells, ultimately causing cell death. Parasites were found to detach from the dead cells. Also, live host cells were preferentially ingested by amebic trogocytosis while pre-killed cells were taken up by phagocytosis. Although the molecular mechanism behind trogocytosis is not well understood, it was shown that the overlapping signaling pathways involving EhC2PK and PI3K, at least partially, regulate both phagocytic and trogocytic processes. Though there are several pathways common to both processes, differences likely exist. Researchers recently attempted to understand how trogocytosis was mechanistically distinct from phagocytosis. AGC family kinase 1 was found to be specifically involved in trogocytosis of live human cells and did not participate in phagocytosis of dead cells [9].

Biological processes equivalent of *E. histolytica* trogocytosis were previously identified in other human pathogens such as *Naegleria fowleri.* However, parasites like *N. fowleri* use a specialized cytoplasmic extension called “amoebostome” or “food-cup” for host cell attachment and ingestion [10]. Structures homologous to *Naegleria* “food-cup” have not been found in *E. histolytica*, suggesting variations in this apparently similar process between organisms.

It appears that not all human epithelial cells are vulnerable to *E. histolytica* trogocytosis to the same levels. In a recent report, LS174T colonic epithelial cells, grown in monolayers, were rarely observed to undergo *E. histolytica*–mediated trogocytosis. The authors postulated that limited flexibility of the plasma membrane due to junctional complexes in cells grown in monolayers might limit trogocytosis by the parasite [11].

## Inflammation and Macrophage Migration Inhibitory Factor Cytokine

Inflammation is a hallmark of amebic colitis; in fact, colitis literally means inflammation of the colon (Fig. 1). Amebic colitis is characterized by the secretion of pro-inflammatory cytokines and infiltration of the colon by immune cells, which would explain why it is often misdiagnosed as inflammatory bowel disease [2•]*.* The gut inflammatory response also contributes to the tissue destruction seen in amebic colitis [12]. This notion was best exemplified in a previous study using a mouse model, which showed that persistent inflammation induced the classic flask-shaped ulcer of amebic colitis despite the lack of significant tissue invasion by *E. histolytica* parasites [13].

Neutrophils are one of the first responders to infiltrate the intestinal tract at the site of inflammation during amebic colitis [12, 14]. Neutrophils are implicated in both protection and pathology in amebic mouse models [15, 16]. Neutrophils generate oxygen free radicals that are capable of killing invading parasites, but are also responsible for collateral tissue damage during the inflammatory period, which might explain their protective and destructive role in amebiasis [12]. This is further compounded by the fact that *E. histolytica* parasites have evolved strategies to evade the immune response and persist in the host, further exacerbating the damage caused by the lingering inflammatory response to invading parasites [12].

Intestinal epithelial cells are the first cell types to come into contact with *E. histolytica* (Fig. 2). It has been known for a while that before making cell contact, trophozoites secrete immunomodulatory proteins that stimulate epithelial cells, resulting in cytokine production and subsequent inflammatory cell infiltration [17, 18]. A variety of cells, including intestinal epithelial cells, produce interleukin-8 (IL-8), a potent neutrophil chemoattractant cytokine. In patients with amebic colitis, higher colonic tissue levels of IL-8 and neutrophils are associated with more severe disease [19–21]. Another cytokine that can result in undesirable effects when produced in excess is TNF-α. Higher TNF-α production was shown to correlate with *E. histolytica* diarrhea in children [22]. TNF-α was also shown to mediate the tissue destruction seen in amebic liver abscess in a mouse model [23].

Many parasitic pathogens, including *E. histolytica*, express a homolog of the pro-inflammatory cytokine macrophage migration inhibitory factor (MIF) [24–28, 29••]. *E. histolytica* MIF (*Eh*MIF) was recently shown to be a crucial parasite mediator of gut inflammation [29••]. A positive correlation was found between *Eh*MIF levels and intestinal inflammation in persons with amebic colitis. *Eh*MIF induced chemokine expression, neutrophil influx, and mucosal damage in cellular and mouse models of amebiasis [29••]. Also, *Eh*MIF enhanced TNF-α production by macrophages [25]. Infected children develop antibodies against *Eh*MIF, which correlates with protection from future infection [25, 29••]. These studies suggest that *Eh*MIF might have a role as an effective immunomodulatory target to prevent or reduce immunopathology in amebiasis.

Inflammasomes are key signaling platforms that detect pathogens and activate the pro-inflammatory cytokines IL-1β and IL-18 [30]. The NLRP3 inflammasome is activated by *E. histolytica*; however, its role in immunopathology and tissue destruction is not yet fully understood [31]*.* While a recent report found that MIF was required for NLRP3 inflammasome activation [32], the role of *Eh*MIF in NLRP3 inflammasome activation remains unknown.

Nitroimidazoles antibiotics, such as metronidazole, are the mainstay therapy for amebic colitis [1••]. However, metronidazole alone is sometimes not enough for severe amebic colitis and even surgical removal of the inflamed colon may or may not prevent death [2•]. Studies in preclinical mouse models show that while metronidazole is very effective at killing ameba, it had little effect on *E. histolytica*–induced mucosal inflammation [33, 34]. Blocking parasite mediators of host inflammation such as *Eh*MIF may help to attenuate disease, and these adjunctive anti-inflammatory strategies may lead to the improvement of the clinical outcomes of amebic colitis.

## Invasion

Tissue invasion by *E. histolytica* plays an important role in the pathogenesis of amebiasis. In clinicopathological studies of patients with severe amebic colitis, lesions extending beyond the cecum and depth of tissue invasion were associated with worse outcomes [35, 36].

The extracellular matrix (ECM) is a complex meshwork of proteins that provides support for tissues. The ECM can act as a physical barrier; thus, breakdown of the ECM is often required to promote cell movement through tissue. Matrix metalloproteinases (MMPs) are the main enzymes involved in ECM degradation, with at least 23 human MMPs identified so far [37].

MMPs are overexpressed in all protozoan infections [38]. In the context of amebiasis, MMPs were among the top 5 most overexpressed genes in colonic tissue from patients suffering from intestinal amebiasis, and they were recently shown to be necessary for invasion. In a human colon explant model, inhibition of MMP activity blocked invasion of colonic mucosa by *E. histolytica* [14, 39••]. *Eh*MIF has been found to influence the levels of MMP production. The inflammation induced by *Eh*MIF resulted in an increase in MMP production [29••]. Therefore, *E. histolytica* parasites may produce MIF to exploit the host inflammatory response to promote tissue invasion.

## Human Gut Microbiome and *E. histolytica* Infection

*E. histolytica* and bacteria have coevolved in the same intestinal environment over evolutionary time. *S*tudies in microbiota-controlled animal models dating back more than half a century ago demonstrated that ulcerative lesions caused by *E. histolytica* were significantly enhanced in the presence of bacteria [40]. The rapid expansion of human microbiome data has created opportunities for further understanding the role of the microbiota in amebiasis.

Recently, several human studies have revealed a significant change in the microbiota of patients with amebiasis. A strong correlation between the presence of *E. histolytica* and certain bacterial groups of the intestinal microbiota was found in an evaluation of the microbiota composition in rural populations of Cameroon [41]. However, this study had several limitations, including the use of microscopy to detect *Entamoeba*. Microscopy does not distinguish nonpathogenic *E. dispar* from *E. histolytica*, the causative agents of amebiasis [1••]. Higher abundance of *Prevotella copri*, a species associated with gut inflammation, was found in children with diarrhea due to *E. histolytica* as compared with those with asymptomatic *E. histolytica* infection in a study conducted in an endemic area of Bangladesh [42••]. Also, a cross-sectional study of patients attending gastroenterology clinics in the Limpopo Province of South Africa found higher levels of *P. copri* in diarrhea due to *E. histolytica* when compared with nondiarrheal *E. histolytica* colonization [43]. While these studies did not determine whether *P. copri* changes were the cause or effect of the disease, preliminary data suggest that *P. copri* expansion occurred prior to infection with *E. histolytica* and symptomatic disease [44].

*Prevotella* species are anaerobic gram-negative bacteria that constitute a major part of the normal flora of the mouth, gastrointestinal tract, and female genital tract. Interestingly, emerging studies have linked increased *Prevotella* abundance to inflammatory disorders [45]. *Prevotella* spp. are capable of inducing IL-8 and IL-6 secretion by epithelial cells, resulting in Th17 responses and neutrophil recruitment in the intestinal mucosa [45]. It is plausible that increased *P. copri* disrupts gut homeostasis and tips the balance toward a pro-inflammatory state which then amplifies *E. histolyica*–induced inflammation, resulting in immunopathology and symptomatic infection. Further studies, however, are needed to confirm this theory.

## Genetic Tools for Investigating *E. histolytica* Pathogenesis

The molecular processes underlying the pathogenicity of *E. histolytica* are not fully understood. Therefore, genetic techniques are needed to further understand the pathogenesis of disease caused by *E*. *histolytica.* Here, we discuss the developments in the genetic methods applicable to *E. histolytica*.

Gene silencing has been achieved using a unique strain of *E. histolytica* called G3, where several amoebapore genes (*ap-a*, *ap-b*, and *SAPLIP*) were permanently silenced [46]. Using this strain and the regulatory region of the endogenous *ap-a* gene, several different genes have been successfully silenced [9]. However, this method does not allow for the silencing of a single gene. Gene silencing by exploiting the endogenous RNA interference pathway of eukaryotic cells has also been utilized in *E. histolytica* [47]. Efficient but variable degrees of gene silencing are achieved by this approach. RNA interference methods that have been used successfully include antisense RNA expression [7] and RNAi “trigger”-based gene silencing [48].

In addition to knocking down gene expression, overexpression is often used to study the impact of gain of gene function in an organism. Different vector systems have been successfully used in *E. histolytica* for constitutive and inducible gene overexpression. For example, using the pKT3M vector, *Eh*MIF gene was constitutively overexpressed in a virulent strain of *E. histolytica*, which was used to study its effect on mucosal inflammation in an amebic colitis mouse model [29••]. It is important to note that *Eh*MIF is a soluble secreted and non-toxic protein which enabled successful constitutive overexpression [29••]. In addition, inducible systems such as pEhHYGB-Tet-0-CAT vector are available for tetracycline-induced overexpression [49]. Furthermore, studies exploring *E. histolytica* virulent genes have overexpressed a dominant negative mutant protein to inhibit the endogenous protein function [9].

Despite all the current methods available for genetic manipulation, there is a need for developing a complete gene knockout system in *E. histolytica.* This has been achieved in other protist parasites [50, 51]. Efforts are underway to develop CRISPR-Cas9-mediated gene targeting in *E. histolytica.* So far, successful expression of the guide RNA and a modified version of Cas9 in *E. histolytica* trophozoites using a single plasmid approach has been achieved.

## Conclusion

*E. histolytica* infection continues to cause great suffering worldwide. Recent studies have helped to expand our understanding of the pathogenesis of amebiasis. The pathogenesis appears to result from parasite cytotoxic activity, damaging inflammatory response, and invasion. Despite these advances, fundamental questions remain unanswered (Table 2). The answers to these questions may lay the foundation for translating scientific discoveries into clinical interventions.

**Table 2:** Outstanding questions

Are there host cell factors that promote resistance to trogocytosis?	
Would nitroimidazoles antiprotozoal therapy in conjunction with anti-inflammatory strategies by blocking *Eh*MIF activity prove superior to nitroimidazoles alone in reducing tissue damage and symptoms in severe amebiasis?	
What is the mechanism by which increased *P. copri* abundance leads to symptomatic *E. histolyica* infection?	
Will CRISPR genetic knockout develop into a reliable technique in *E. histolyica?*	
